# Cardiac Tamponade Secondary to Severe Uremic Pericarditis in the Modern Dialysis Era: A Case Report

**DOI:** 10.7759/cureus.111126

**Published:** 2026-06-19

**Authors:** Piotr Majewski, Zuzanna Jakubowska, Michal Pyrza, Jolanta Malyszko

**Affiliations:** 1 Nephrology, Dialysis and Internal Medicine, Medical University of Warsaw, Warsaw, POL

**Keywords:** cardiac tamponade, end stage renal disease (esrd), heart failure, uremic pericarditis, dialysis

## Abstract

Uremic pericarditis is currently an uncommon complication of end-stage renal disease (ESRD) in developed countries due to improved access to renal replacement therapy and earlier initiation of hemodialysis. Nevertheless, delayed or interrupted dialysis may still result in life-threatening manifestations, including massive pericardial effusion and cardiac tamponade.

We present the case of a 44-year-old man with ESRD requiring hemodialysis, insulin-dependent type 2 diabetes mellitus, morbid obesity, hypertension, and heart failure with preserved ejection fraction who was admitted because of progressive dyspnea, generalized weakness, and systemic deterioration. Following the recent initiation of hemodialysis, renal replacement therapy was interrupted because of recurrent dialysis catheter dysfunction and discontinuation of dialysis care at an outside center.

On admission, the patient presented in moderate-to-severe clinical condition with profound fluid overload estimated at approximately 30 liters above dry weight, respiratory distress, an inflammatory response, severe azotemia, and metabolic acidosis. Physical examination was diagnostically challenging because diffuse pulmonary crackles, wheezes, and coarse breath sounds obscured potential pericardial friction rub, while morbid obesity limited assessment of jugular venous distention.

Chest radiography demonstrated marked cardiomegaly with a globular “water bottle” configuration suggestive of massive pericardial effusion. Subsequent transthoracic echocardiography revealed a large circumferential pericardial effusion with echocardiographic signs of impending cardiac tamponade.

Management included restoration and intensification of renal replacement therapy with repeated hemodialysis sessions and isolated ultrafiltration (IUF), broad-spectrum antimicrobial therapy, and multidisciplinary intensive care treatment. Pericardial fluid cultures remained sterile despite concomitant bloodstream infection associated with a severely contaminated dialysis catheter. Gradual clinical improvement was achieved with the reduction of hypervolemia and stabilization of hemodynamic status.

This case highlights a rare but severe presentation of advanced uremic pericarditis occurring after interruption of renal replacement therapy. Despite being considered a largely historical complication in developed healthcare systems, severe uremic pericarditis should remain in the differential diagnosis in patients with ESRD presenting with progressive dyspnea, cardiomegaly, massive fluid overload, and hemodynamic instability.

## Introduction

Uremic pericarditis is a historically recognized complication of advanced kidney failure that has become increasingly uncommon in developed countries following widespread availability of renal replacement therapy and earlier initiation of hemodialysis. Before the modern dialysis era, it represented one of the most severe cardiovascular manifestations of untreated uremia, frequently progressing to large pericardial effusions and cardiac tamponade. Contemporary nephrology practice has markedly reduced its incidence, making severe uremic pericarditis an increasingly rare clinical entity. The actual incidence of uremic pericarditis is difficult to determine due to variability in clinical presentation and diagnostic criteria. Recent studies have shown that its prevalence has declined to below 5% in recent decades [1].

The cause of uremic pericarditis is primarily related to the accumulation of toxic metabolites and nitrogenous waste in the blood, due to end-stage renal failure, triggering a systemic and local pro-inflammatory state [1]. The most common symptoms of uremic pericarditis are pleuritic chest pain that worsens in the supine position, fever, chills, malaise, dyspnea, and cough.

Pericarditis in patients with end-stage renal disease is traditionally divided into uremic pericarditis and dialysis pericarditis. Uremic pericarditis is defined as occurring in patients who have not undergone renal replacement therapy or as occurring within the first eight weeks of initiating therapy. Dialysis pericarditis, in turn, is defined as the onset of symptoms after eight weeks from the start of renal replacement therapy. However, patients already receiving dialysis are also at risk of uremic pericarditis due to inadequate dialysis technique or non-adherence to renal replacement therapy [2].

Despite these advances, interruption or inadequacy of dialysis treatment may still lead to rapid accumulation of uremic toxins, profound fluid overload, and severe inflammatory complications. Delayed access to dialysis, vascular access dysfunction, fragmented continuity of care, and dialysis nonadherence remain important risk factors for severe uremic manifestations even in developed healthcare systems.

Diagnosis may be particularly challenging in critically ill patients with coexisting infection, heart failure, severe hypervolemia, and respiratory compromise, as symptoms are often nonspecific and overlap with manifestations of multiorgan dysfunction. Furthermore, classic physical examination findings associated with cardiac tamponade may be obscured in patients with morbid obesity and extensive pulmonary abnormalities.

We report a rare contemporary case of severe uremic pericarditis complicated by massive hemorrhagic pericardial effusion and impending cardiac tamponade following interruption of hemodialysis caused by recurrent dialysis catheter dysfunction and discontinuity of dialysis care. The patient presented with extreme hypervolemia estimated at approximately 30 liters above dry weight and required intensified renal replacement therapy, including repeated hemodialysis and isolated ultrafiltration procedures.

## Case presentation

A 44-year-old man with a history of end-stage renal disease (ESRD) requiring hemodialysis, insulin-dependent type 2 diabetes mellitus, morbid obesity, hypertension, heart failure with preserved ejection fraction (HFpEF), chronic pancreatitis, and right lower extremity amputation secondary to diabetic foot syndrome was admitted to the Department of Nephrology because of progressive dyspnea, generalized weakness, lower extremity edema, and significant deterioration of overall functional status.

Several weeks prior to admission, the patient had initiated intermittent hemodialysis due to advanced kidney failure. However, continuation of renal replacement therapy was interrupted for approximately 1.5 weeks because of recurrent dialysis catheter dysfunction. Additionally, the patient discontinued treatment at an outside dialysis center following interpersonal conflict with medical personnel, resulting in substantial interruption of dialysis care.

Before admission, the patient reported progressive exercise intolerance, orthopnea, worsening peripheral edema, and marked fatigue. On presentation, he was in moderate-to-severe clinical condition with a heart rate of 101 bpm, tachypnea, blood pressure of 112/67 mmHg, and features of profound hypervolemia estimated at approximately 30 liters above dry weight. Physical examination demonstrated generalized edema, bilateral pulmonary crackles, diffuse wheezes, coarse breath sounds, and signs of severe fluid overload. Cardiovascular examination was diagnostically challenging because potential pericardial friction rub was obscured by extensive pulmonary findings, while assessment of jugular venous distention was limited by morbid obesity.

Inspection of the tunneled dialysis catheter revealed marked external contamination with inflammatory changes around the insertion site, raising suspicion of catheter-related bloodstream infection. During the initial phase of hospitalization, the patient additionally required naloxone administration because of suspected buprenorphine overexposure related to transdermal analgesic therapy used for chronic pain management, further complicating neurological and clinical assessment.

Initial laboratory investigations demonstrated severe metabolic derangement with serum urea concentration reaching 235 mg/dL, creatinine levels exceeding 10 mg/dL, metabolic acidosis, normocytic anemia, hypoalbuminemia, elevated N-terminal pro-B-type natriuretic peptide (NT-proBNP) concentrations, and markedly increased inflammatory markers, including C-reactive protein (CRP) levels above 220 mg/L; troponins were within the reference range (Table 1).

**Table 1:** Key laboratory parameters on admission demonstrating severe metabolic derangement associated with advanced untreated uremia and profound hypervolemia. NT-proBNP: N-terminal pro–B-type natriuretic peptide; CRP: C-reactive protein; INR: International Normalized Ratio; aPTT: activated partial thromboplastin time.

	Value on admission	Reference range
Hemoglobin	8.1 g/dL	13.5–17.5 g/dL
Serum urea	235 mg/dL	15–40 mg/dL
Creatinine	10.1 mg/dL	0.7–1.3 mg/dL
Potassium	5.9 mmol/L	3.5–5.1 mmol/L
pH	7.10	7.35–7.45
Bicarbonate (HCO₃⁻)	15 mmol/L	22–28 mmol/L
NT-proBNP	10707 pg/mL	<125 pg/mL
CRP	225 mg/L	<5 mg/L
Troponin-I	5 ng/L	<60 ng/L
INR	1.29	0.8-1.2
aPTT	30.9 s	25-37 s

**Figure 1:**
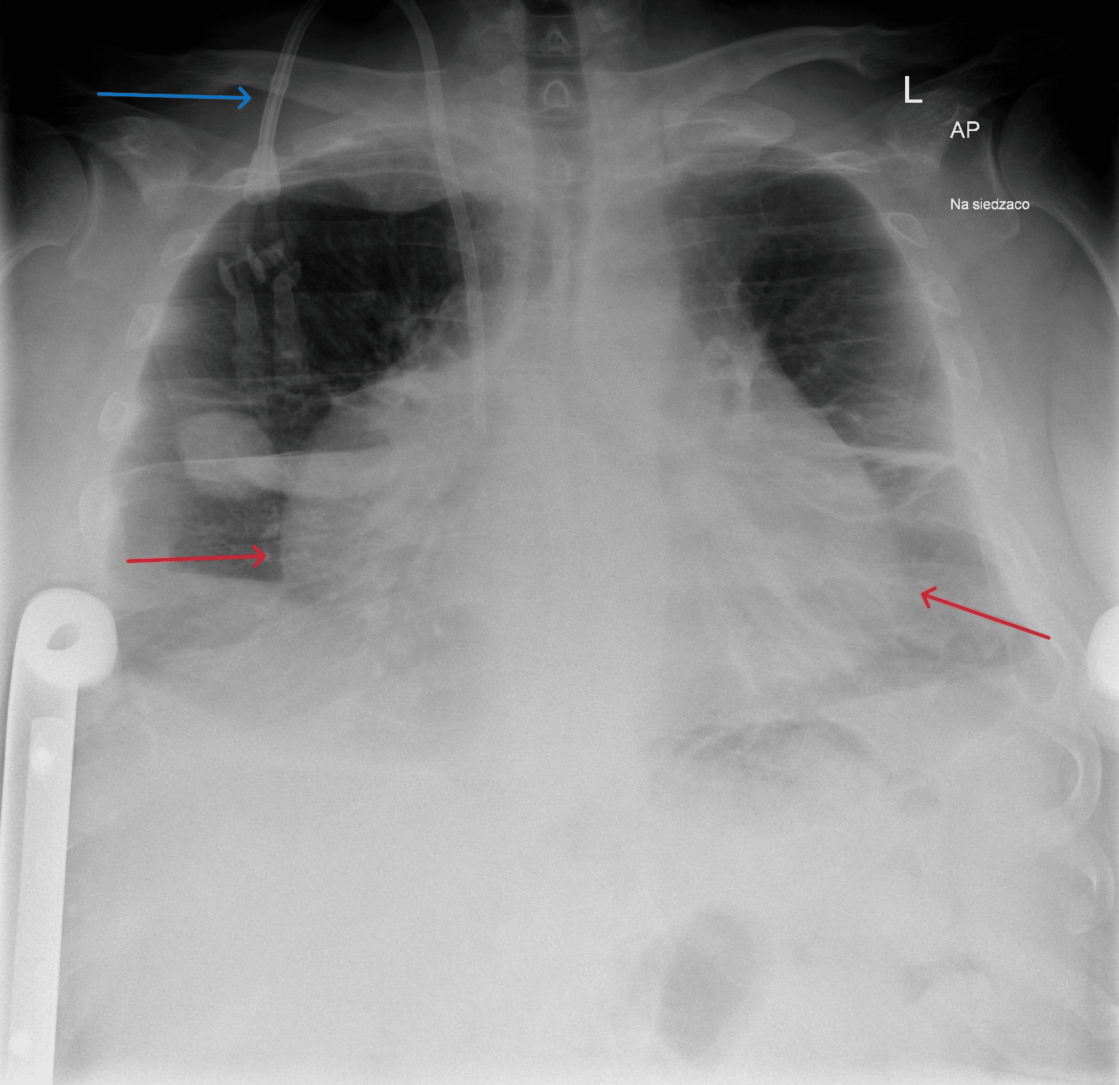
Chest radiograph obtained on admission. Demonstrated marked enlargement of the cardiac silhouette with a globular “water bottle” configuration consistent with massive pericardial effusion (red arrows). Additional findings included pulmonary vascular congestion and bilateral pleural effusions in the setting of profound hypervolemia secondary to interrupted hemodialysis. A tunneled permanent dialysis catheter is visible on the right side, inserted via the right internal jugular vein (blue arrow).

Blood and catheter-associated cultures yielded multidrug-resistant organisms, including Klebsiella pneumoniae oxacillinase (OXA)-positive strains and Staphylococcus epidermidis. Review of chest radiographs obtained during current and previous hospitalizations demonstrated progressive and marked enlargement of the cardiac silhouette with a characteristic globular “water bottle” appearance suggestive of massive pericardial effusion (Figure 1). Recognition of the disproportionate cardiomegaly prompted urgent transthoracic echocardiography.

**Figure 2:**
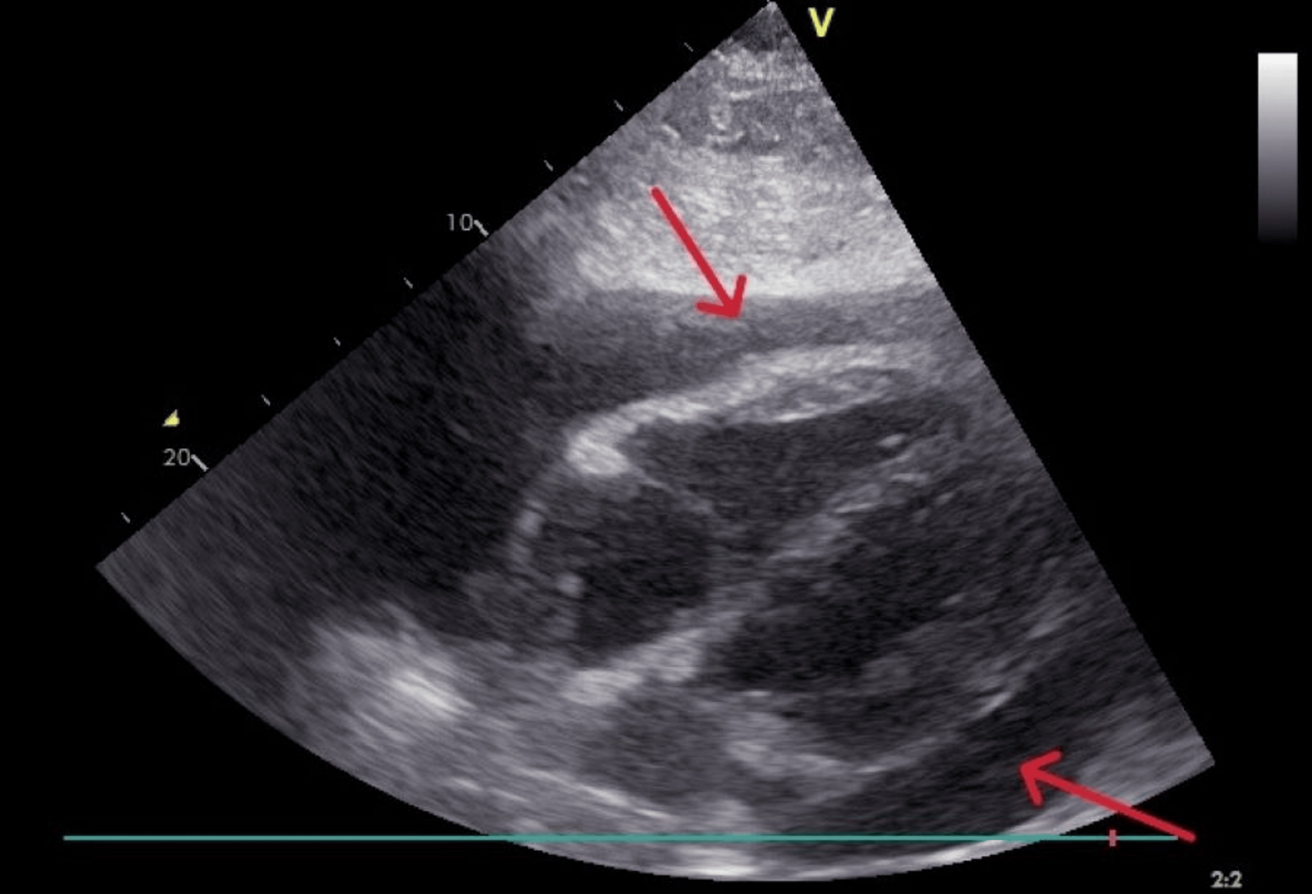
Transthoracic echocardiography image shows a significant volume pericardial fluid (red arrows).

Echocardiographic examination revealed a large circumferential pericardial effusion with hemodynamically significant features of impending cardiac tamponade, including right ventricular diastolic collapse, respiratory variation of mitral inflow, and a dilated non-collapsing inferior vena cava. Pericardial fluid measurements reached approximately 20 mm posteriorly, 25 mm laterally, and 23 mm adjacent to the right ventricle (Figure 2, Video 1, Table 2).

**Video 1:** Transthoracic echocardiography video shows right ventricular diastolic collapse and pericardial fluid.

**Table 2:** Echocardiographic parameters. BSA: body surface area; LV: left ventricular internal diameter; IVS: interventricular septal thickness; PW: posterior wall thickness; LVM: left ventricular mass; LVMI: left ventricular mass index; EF 4Ch: left ventricular ejection fraction measured in the apical four-chamber view; LA: left atrial diameter; LAVI: left atrial volume index; RV4Ch: right ventricular basal diameter in the apical four-chamber view; AO r: aortic root diameter; AO asc: ascending aortic diameter; IVC: inferior vena cava diameter.

Parameter	Value	Reference range
Weight [kg]	118 kg	
Height [cm]	180 cm	
BSA	2.35	
LV [mm]	45 mm	<59 mm
LV [mm/m^2^]	18.29 mm/m^2^	<31 mm/m^2^
AO r [mm]	35 mm	≤45 mm
AO r [mm/m^2^]	14.23 mm/m^2^	<20 mm/m^2^
AO asc [mm/m^2^]	0 mm/m^2^	<17 mm/m^2^
LA [mm]	48 mm	≤40 mm
LA [mm/m^2^]	19.51 mm/m^2^	≤23 mm/m^2^
RV4ch [mm]	39 mm	<42 mm
IVS [mm]	16 mm	6-10 mm
PW [mm]	15 mm	6-10 mm
LVMI [g/m^2^]	117.65 g/m^2^	≤115 g/m^2^
LAVI [ml/m^2^]	0 ml/m^2^	≤34 ml/m^2^
LVM [g]	196.92	
EF 4ch [%]	60 %	55–70%
IVC [mm]	26 mm	≤20 mm

The patient was urgently transferred to the intensive care unit, where emergent pericardiocentesis was performed with evacuation of approximately 1200 mL of hemorrhagic pericardial fluid and placement of a pericardial drain. Pericardial fluid analysis demonstrated exudative hemorrhagic characteristics (Table 3). In a cytological examination, no malignant cells were found; among abundant blood morphological elements, a few neutrophils, and activated mesothelial cells were observed. A markedly elevated concentration of urea was found in the pericardial fluid, confirming the diagnosis of uremic pericarditis. Importantly, pericardial fluid cultures remained sterile despite concomitant bloodstream infection.

**Table 3:** Pericardial fluid analysis. The pericardial fluid had the characteristics of an exudate mixed with blood. RBC: red blood cells; WBC: white blood cells; LDH: lactate dehydrogenase; CRP: C-reactive protein.

Parameter	Value	Comments	Reference range
Sample volume	110 ml		
Color	red		
Fluid clarity	cloudy		
pH	7.12		
Specific gravity	1.024 g/cm^3^		
Total nucleated cell count	4459 cells/µL		
RBC	0,694×10^6^ cells/µL		
WBC	4446 cells/µL		
Neutrophils	80%		
Eosinophils	4%		
Lymphocytes	7%		
Monocytes	9%		
Total protein in pericardial fluid	5.5 g/dL		
Total protein in serum	6.2 g/dL		6.2-8.2 g/dL
Ratio of protein concentration in pericardial fluid to protein concentration in blood serum	0.89	<0.5 Transudate >0.5 Exudate	
LDH in fluid	495 U/L		
LDH in serum	197 U/L		135-225 U/L
Ratio of LDH activity in pericardial fluid to LDH activity in serum	2.51	<0.6 Transudate >0.6 Exudate	
Glucose	116 mg/dL		
Lactate in pericardial fluid	4,2 mmol/L		
Urea in pericardial fluid	102 mg/dL		
CRP in pericardial fluid	72 mg/L		

Additional diagnostic workup was performed to evaluate alternative etiologies of the pericardial effusion. Interferon-gamma release assay testing using Quantiferon (QIAGEN, Venlo, The Netherlands) was negative, reducing the likelihood of tuberculous pericarditis. Autoimmune screening, including antinuclear antibodies (ANA), was also negative.

Management included restoration and intensification of renal replacement therapy with repeated hemodialysis sessions combined with isolated ultrafiltration (IUF) procedures to achieve gradual correction of profound hypervolemia and metabolic instability. During the first week of hospitalization, the patient underwent daily hemodialysis sessions combined with isolated ultrafiltration (IUF). Due to persistent intradialytic hypotension occurring during every dialysis session in the first week, aggressive fluid removal was not feasible. Therefore, ultrafiltration was performed gradually, with approximately 1000-1500 mL removed during each hemodialysis/IUF session. Subsequently, dialysis treatments were continued daily (except Sundays) to achieve progressive correction of the estimated 30-liter fluid overload. No dialysis disequilibrium syndrome was observed during hospitalization. Broad-spectrum antimicrobial therapy was administered because of concomitant catheter-related bloodstream infection and sepsis.

**Figure 3:**
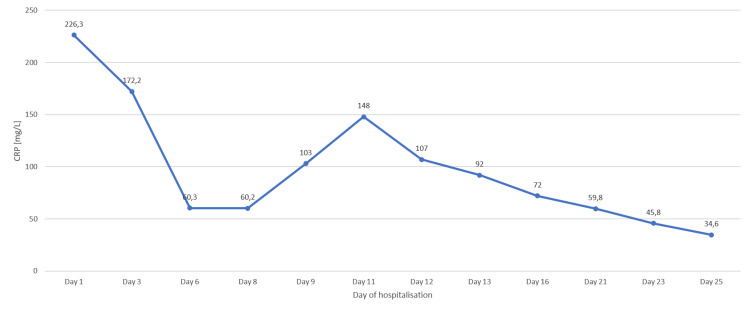
The graph illustrates changes in C-reactive protein (CRP) levels over the course of hospitalization.

**Figure 4:**
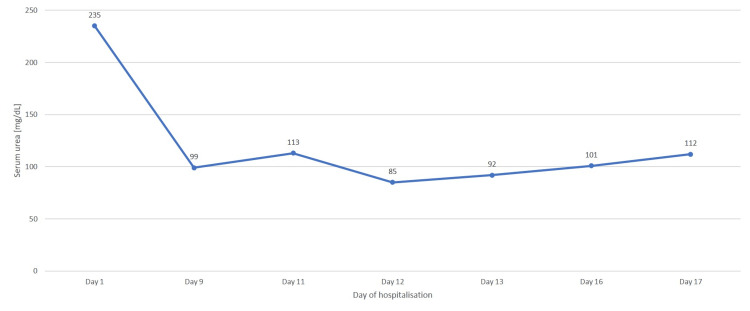
The graph illustrates the changes in serum urea concentration over the course of hospitalization.

Over subsequent days, progressive clinical improvement was observed, including reduction of dyspnea, improvement in respiratory status, and gradual resolution of volume overload. A decline in inflammatory markers and urea concentration was observed (Figures 3, 4). Serial echocardiographic examinations demonstrated a significant reduction of pericardial effusion without recurrence of tamponade physiology (Table 4). The patient was ultimately discharged in improved general condition with recommendations for continuation of chronic hemodialysis and close nephrology and cardiology follow-up.

**Table 4:** The table illustrates the changes in pericardial effusion volume across serial echocardiographic studies.

Number of echocardiographies	Findings
First echocardiography	Pericardial fluid measurements reached approximately 20 mm posteriorly, 25 mm laterally, and 23 mm adjacent to the right ventricle
Second echocardiography was performed the day after the first echocardiography	Pericardial fluid up to 27 mm from the posterior wall of the left ventricle, and up to 11 mm from the right ventricle.
Third echocardiography was performed four days after the first echocardiography	Pericardial fluid up to 16 mm from the posterior wall of the left ventricle and up to 9 mm from the right ventricle
Fourth echocardiography was performed four days after the first echocardiography	Pericardial fluid up to 21 mm from the posterior wall of left ventricle.
Fifth echocardiography was performed eighteen days after the first echocardiography, before hospital discharge.	A significantly smaller amount of pericardial fluid is present, measuring 15 mm adjacent to the left ventricle and 5 mm adjacent to the right ventricle. The pericardial effusion is not hemodynamically significant.

Two years later, the patient was rehospitalized at our center. Follow-up echocardiography showed a non-dilated left ventricle with a left ventricular ejection fraction of 55%, no significant valvular abnormalities, normal right ventricular systolic function, a non-collapsible inferior vena cava, and a normal pericardium without evidence of effusion. The patient had been attending hemodialysis sessions regularly.

## Discussion

Severe uremic pericarditis has become an increasingly uncommon clinical entity in developed countries due to the widespread availability of renal replacement therapy and earlier initiation of dialysis in patients with ESRD [1,3]. The probable cause of uremic pericarditis is inflammation of the parietal and visceral layers of the pericardium due to the accumulation of toxic metabolites, blood urea nitrogen, and serum creatinine [4]. Historically, however, uremic pericarditis represented one of the most serious cardiovascular complications of untreated uremia and was associated with substantial mortality, particularly after progression to cardiac tamponade [2].

In the present case, interruption of hemodialysis appeared to represent the pivotal pathophysiological event leading to progressive accumulation of uremic toxins, profound hypervolemia, and subsequent development of severe hemorrhagic pericardial effusion. In this case, prolonged interruption of dialysis resulted in rapid progression of severe metabolic and hemodynamic complications. severe uremic pericarditis should remain in the differential diagnosis. At admission, severe metabolic derangement was evident, including marked azotemia with serum urea concentration reaching 235 mg/dL, creatinine levels exceeding 10 mg/dL, metabolic acidosis, and extreme fluid overload estimated at approximately 30 liters above dry weight.

The coexistence of sepsis and multidrug-resistant bloodstream infection substantially complicated the diagnostic process. The patient presented with markedly elevated inflammatory markers, positive microbiological cultures, and systemic inflammatory response syndrome. Nevertheless, several clinical findings supported severe uremic pericarditis as the principal mechanism underlying the massive pericardial effusion. These included prolonged interruption of dialysis therapy, profound biochemical uremia, severe hypervolemia, hemorrhagic exudative pericardial fluid, negative pericardial fluid cultures, and significant clinical improvement following restoration and intensification of renal replacement therapy.

Differential diagnosis of large hemorrhagic pericardial effusion in patients with ESRD remains clinically challenging, particularly in the presence of concomitant sepsis and elevated inflammatory markers [2,3,5]. In the present case, purulent bacterial pericarditis was initially considered because of positive blood cultures and suspected catheter-related bloodstream infection.

However, pericardial fluid cultures remained sterile despite positive bloodstream microbiology. The broad-spectrum antibiotic therapy administered previously in response to positive blood cultures may have resulted in sterile pericardial fluid cultures. Furthermore, additional investigations, including Quantiferon testing and autoimmune screening with antinuclear antibodies, were negative, making tuberculous and autoimmune pericarditis less likely. Taken together with the profound biochemical uremia, interruption of dialysis therapy, severe hypervolemia, and favorable response to intensified renal replacement therapy, these findings strongly supported severe uremic pericarditis as the principal mechanism underlying the development of cardiac tamponade.

Hemorrhagic pericardial effusion is a recognized feature of advanced uremic pericarditis and is thought to result from severe inflammation combined with uremia-associated platelet dysfunction and impaired hemostasis [3,6]. In this patient, pericardial fluid analysis demonstrated exudative characteristics with hemorrhagic admixture but without microbiological evidence of purulent infection.

Another clinically important aspect of the present case was the relative obscuration of classic tamponade findings. Physical examination was significantly complicated by morbid obesity, profound hypervolemia, diffuse pulmonary crackles and wheezes, concomitant sepsis, and opioid-related altered mental status. As a result, characteristic findings such as pericardial friction rub and jugular venous distention were difficult to assess reliably. Persistent hypotension and tachycardia ultimately represented the most prominent hemodynamic indicators of impending tamponade. This case, therefore, illustrates how recognition of cardiac tamponade in critically ill dialysis patients may be substantially delayed when multiple overlapping cardiopulmonary abnormalities coexist.

An important diagnostic clue in the present case was the striking enlargement of the cardiac silhouette visible on serial chest radiographs. The globular “water bottle” configuration of the heart, historically associated with large pericardial effusions, prompted urgent echocardiographic evaluation and ultimately led to recognition of impending cardiac tamponade [3,7]. Despite the widespread availability of advanced imaging modalities, this case highlights the continuing diagnostic value of conventional chest radiography in critically ill dialysis patients with nonspecific cardiopulmonary symptoms.

Current management of severe uremic pericarditis primarily relies on urgent initiation or intensification of renal replacement therapy, while pericardiocentesis is indicated in patients with hemodynamic compromise or tamponade physiology [3,4,6,8,9]. In addition to emergent pericardiocentesis, the patient required repeated hemodialysis sessions combined with isolated ultrafiltration procedures because of profound hypervolemia and ongoing metabolic instability.

Gradual reduction of fluid overload and progressive clinical improvement following aggressive dialysis-based management further supported the central role of severe uremia in the pathogenesis of the pericardial disease.

Although severe uremic complications are now rarely encountered in developed healthcare systems, they may still occur in the setting of vascular access dysfunction, fragmented continuity of care, and dialysis nonadherence [10].

## Conclusions

Severe uremic pericarditis remains a potentially life-threatening but increasingly rare complication of ESRD in developed countries. Interruption of renal replacement therapy may rapidly result in profound metabolic derangement, massive pericardial effusion, and cardiac tamponade. This case demonstrates that severe uremic pericarditis should still be considered in dialysis-dependent patients presenting with marked hypervolemia, cardiomegaly, and unexplained hemodynamic compromise, particularly in the setting of interrupted dialysis therapy. Early echocardiographic assessment, pericardiocentesis, and intensified renal replacement therapy may be lifesaving.
